# Novel use of an endoscopic morcellator to assist in removal of a fully embedded esophageal stent

**DOI:** 10.1016/j.vgie.2023.03.010

**Published:** 2023-05-11

**Authors:** Devika Gandhi, Paul Leonor, Wasseem Skef

**Affiliations:** Division of Gastroenterology & Hepatology, Loma Linda University Medical Center, Loma Linda, California

## Abstract

Video 1 Use of endoscopic morcellator to assist in removal of stent.

Use of endoscopic morcellator to assist in removal of stent.

## Background

Complete embedding of self-expandable metal stents is an uncommon adverse event. Granulation tissue formation depends on a variety of factors including stent type, stent size, radial and axial force, and most importantly, stent duration. Endoscopic removal can be challenging and carries risk of bleeding and perforation.^1^ Several methods can be employed, including stent-in-stent technique,^2^ cryoablation,^3^ and argon plasma coagulation,^4^ and even a case of submucosal dissection has been reported.^5^ We report a novel method for removing an embedded esophageal self-expandable metal stent using EndoRotor endoscopic powered resection (Interscope Medical, Inc, Worcester, Mass, USA; Video 1, available online at www.videogie.org).

## Case Presentation

**Figure 1:**
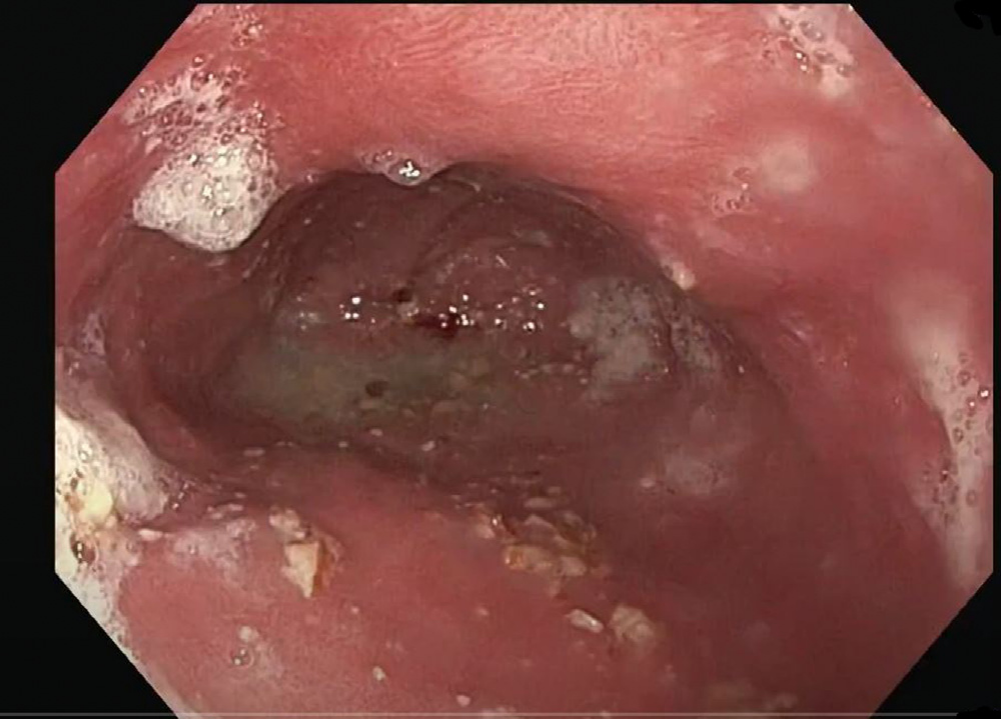
Initial endoscopy demonstrating severe stenosis with completely embedded esophageal stent.

**Figure 2:**
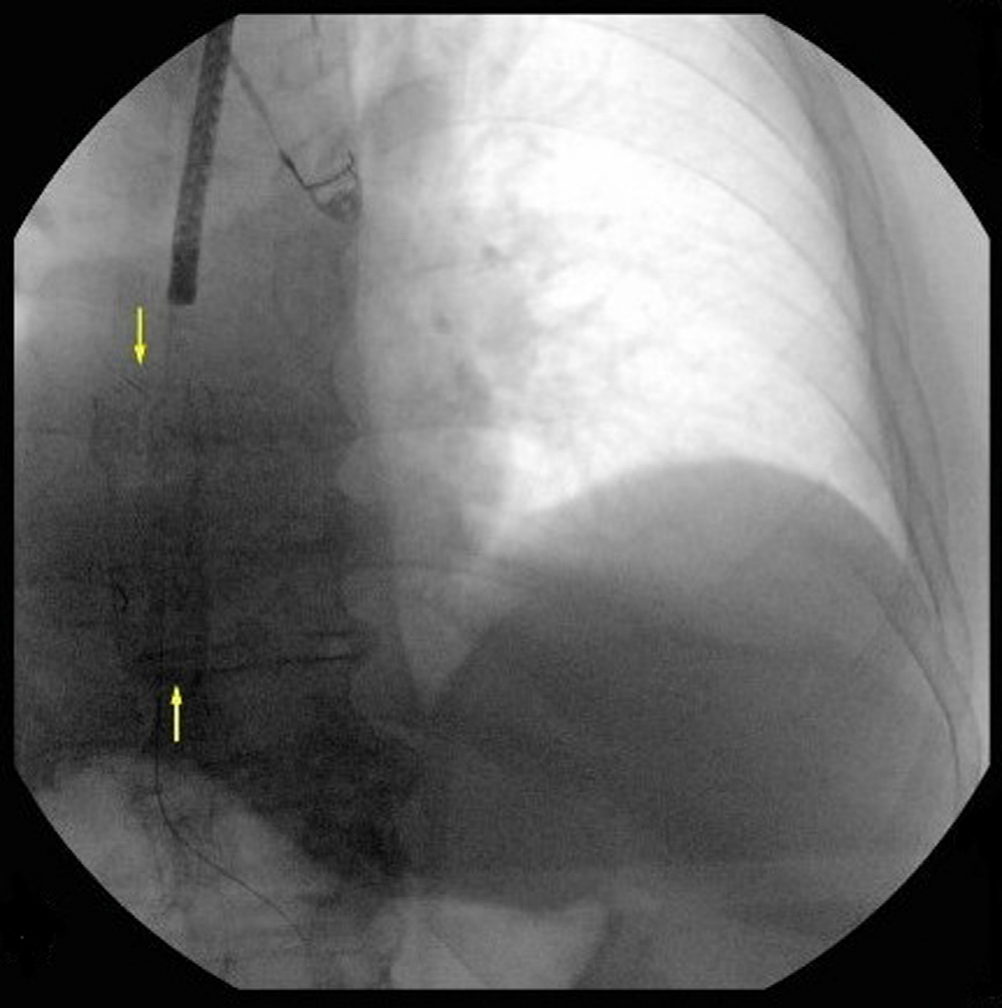
Stent visualized on fluoroscopy.

A 66-year-old man with chronic, refractory benign gastroesophageal junction stricture treated 2 years prior with an 18- × 10-mm Bonastent (Thoracent, Huntington, NY, USA) fully covered self-expandable metal stent (FC-SEMS) presented for progressive dysphagia to both solids and liquids. The patient was lost to follow-up during the COVID-19 pandemic after FC-SEMS placement. EGD revealed a fully embedded esophageal stent with severe stenosis in the distal esophagus with complete tissue ingrowth that was dilated with a Savary dilator (Cook Medical, Bloomington, Ind, USA) to 12.8 mm (Figs. 1 and 2). The patient was initially referred to thoracic surgery for esophagectomy; however, he sought a second opinion at our center for possible endoscopic removal of the embedded stent.

**Figure 3:**
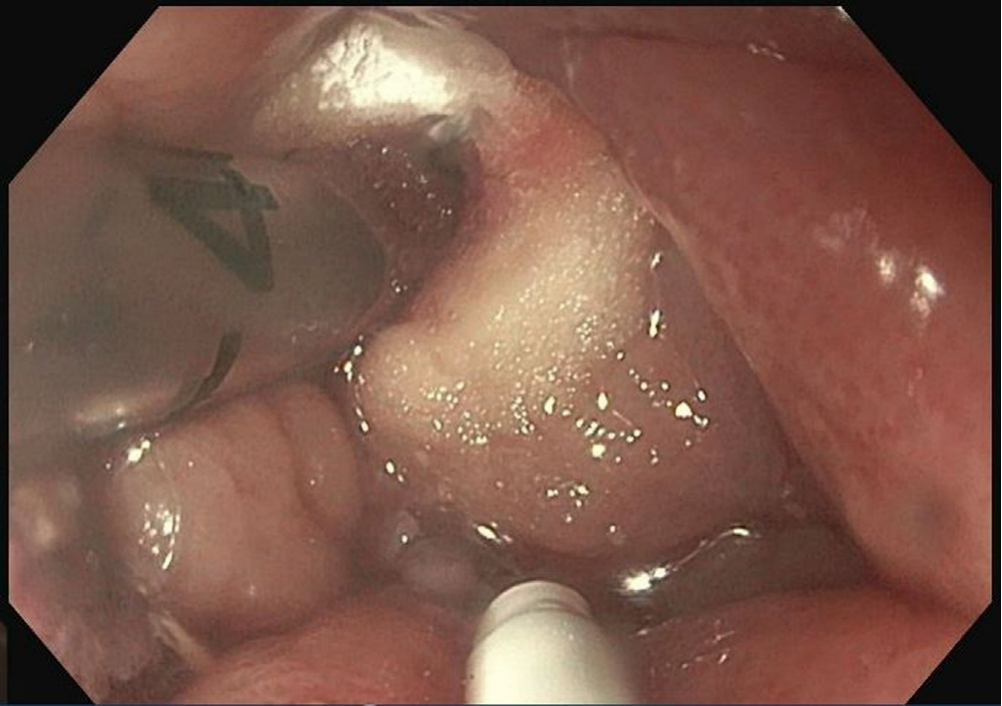
Cryotherapy of tissue ingrowth.

**Figure 4:**
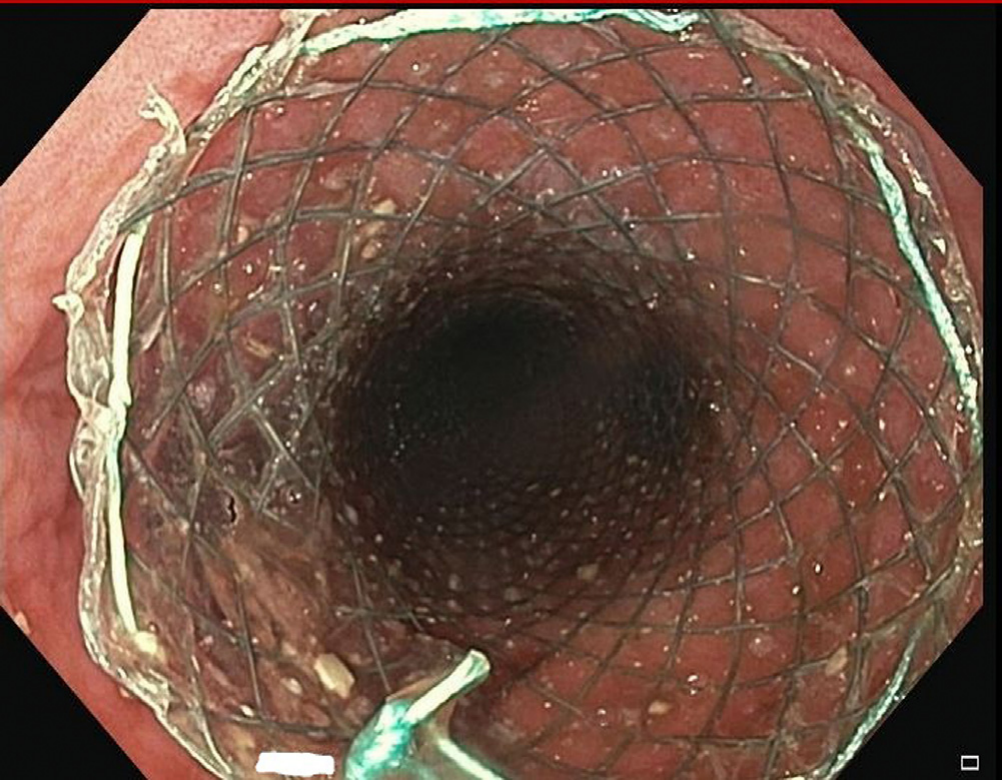
Stent-in-stent technique.

**Figure 5:**
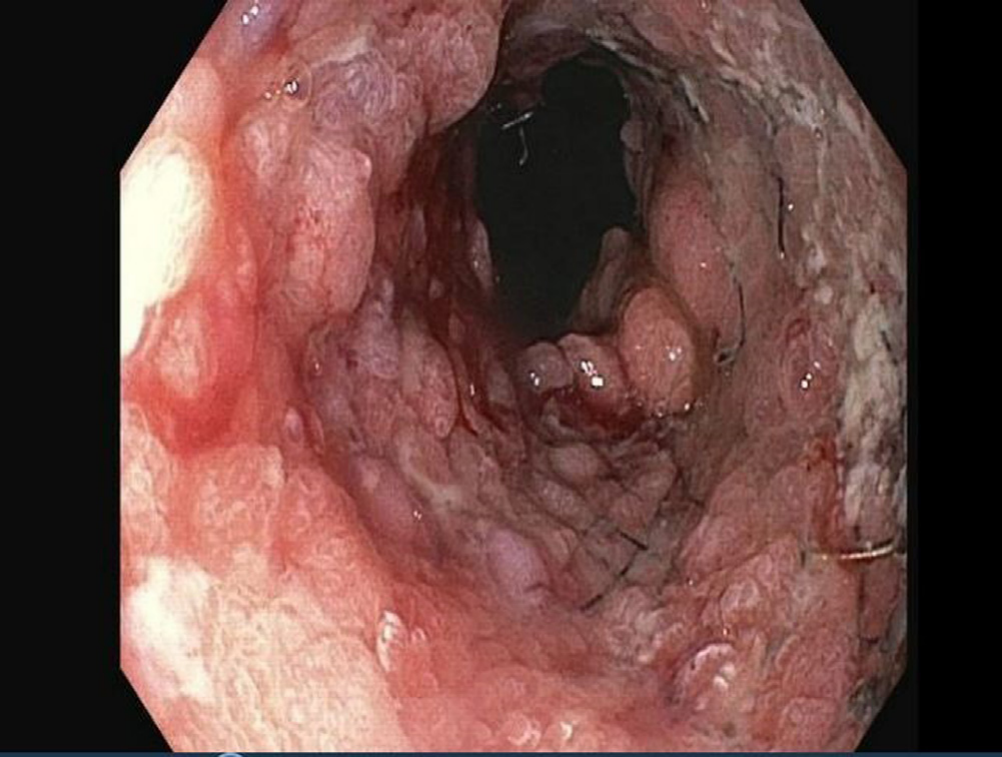
Ongoing tissue ingrowth despite dual modality of cryoablation and stent-in-stent technique.

**Figure 6:**
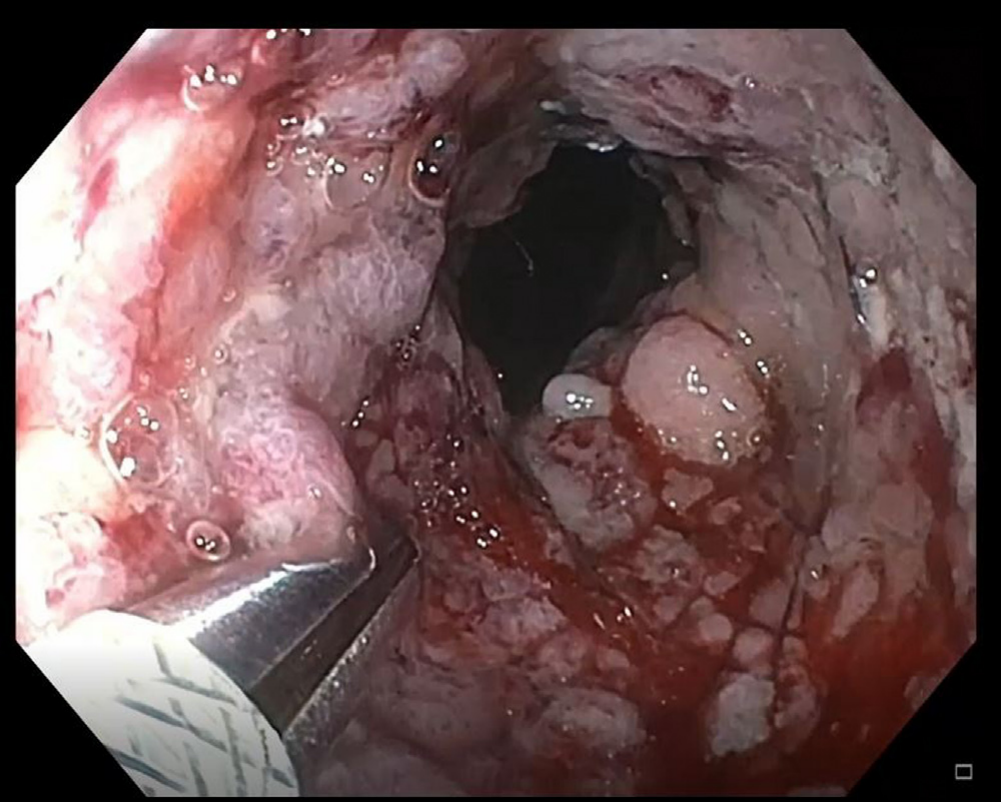
Use of endoscopic morcellator for removal of tissue ingrowth.

**Figure 7:**
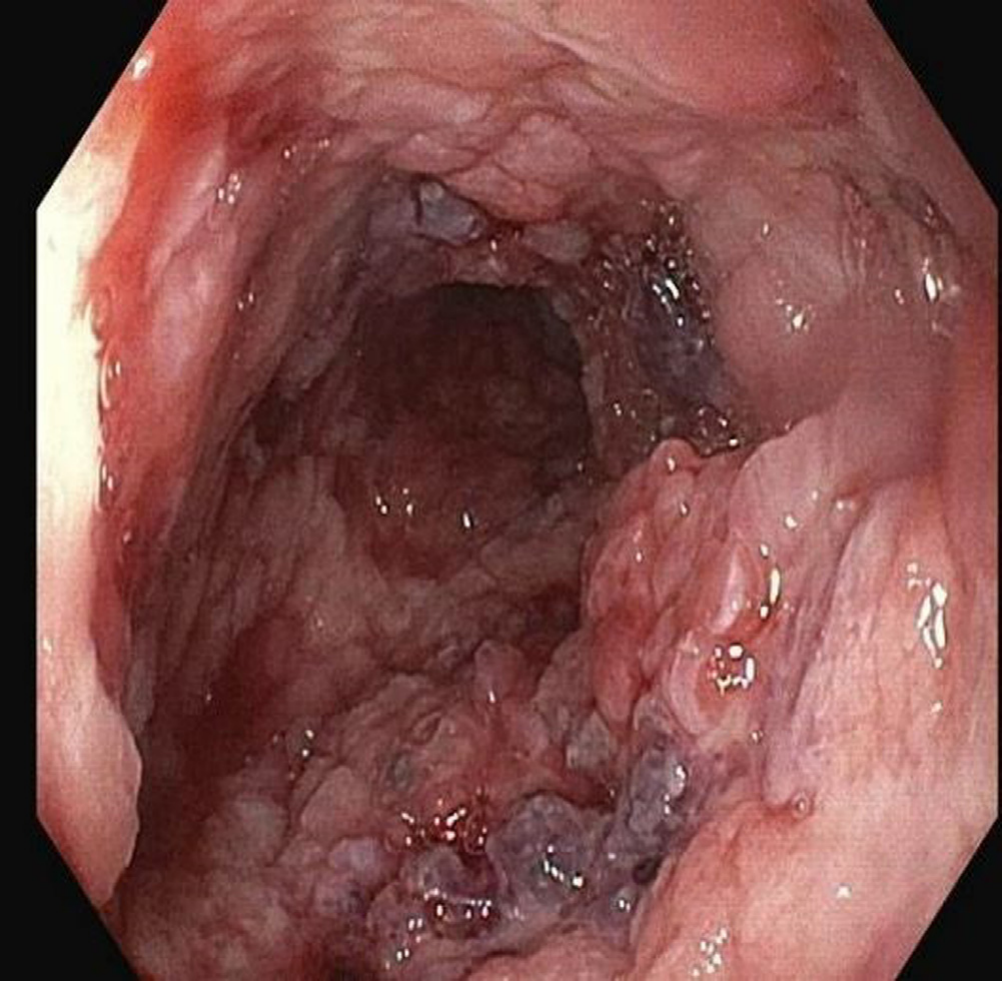
Esophagus immediately postretrieval of embedded stent.

Initially, a combined approach of cryotherapy and stent-in-stent technique with a coaxial 23-mm × 15-cm WallFlex FC-SEMS (Boston Scientific, Boston, Mass, USA) was used to allow for tissue ingrowth necrosis. The patient returned every 4 weeks for repeat endoscopic management for 3 additional sessions (Figs. 3 and 4). Despite our approach, there was still significant tissue ingrowth preventing safe removal of the embedded esophageal stent (Fig. 5). Given insufficient necrosis of granulation tissue, EndoRotor endoscopic-powered resection therapy was used. After removal of the coaxial WallFlex FC-SEMS, 1:10,000 epinephrine was injected into the tissue ingrowth areas for prophylactic hemostasis, and a gastroscope length 3.1-mm endoscopic-powered resection catheter (vacuum setting 50-150 mm Hg, vacuum flow rate 50L/min, and blade speed 1750 RPM) was used to debride the tissue ingrowth with about 95% of the visible granulation tissue removed (Fig. 6). After debridement of granulation tissue, a coaxial FC-SEMS was placed and the patient was discharged home. The duration of the procedure was 70 minutes. On repeat procedure 4 weeks later, removal of the coaxial stent demonstrated that about 75% of the embedded stent was visible. Using the same technique, catheter, and settings, we used the EndoRotor to remove the remaining tissue, which allowed for successful removal of the embedded stent after using rat-toothed forceps to loosen the stent from the wall of the esophagus in several areas. The duration of the second EndoRotor procedure was 89 minutes. Careful inspection of the esophagus confirmed no bleeding or perforation (Fig. 7). Afterward a Boston Agile 18-mm × 10-cm FC-SEMS (Boston Scientific) was deployed under endoscopic guidance traversing the gastroesophageal junction and covering the distal esophagus. The patient was discharged home the same day on a stent diet and maintained on omeprazole twice daily before breakfast and dinner.

**Figure 8:**
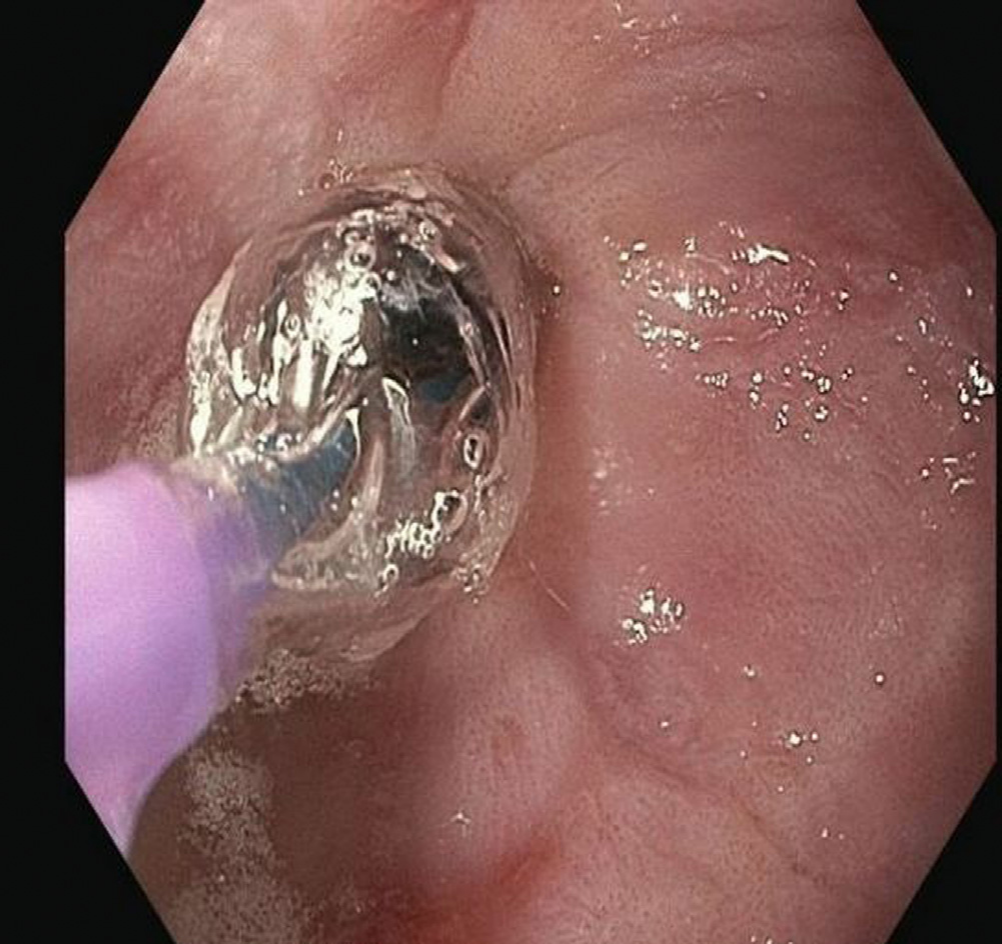
Transendoscopic dilation of stricture.

**Figure 9:**
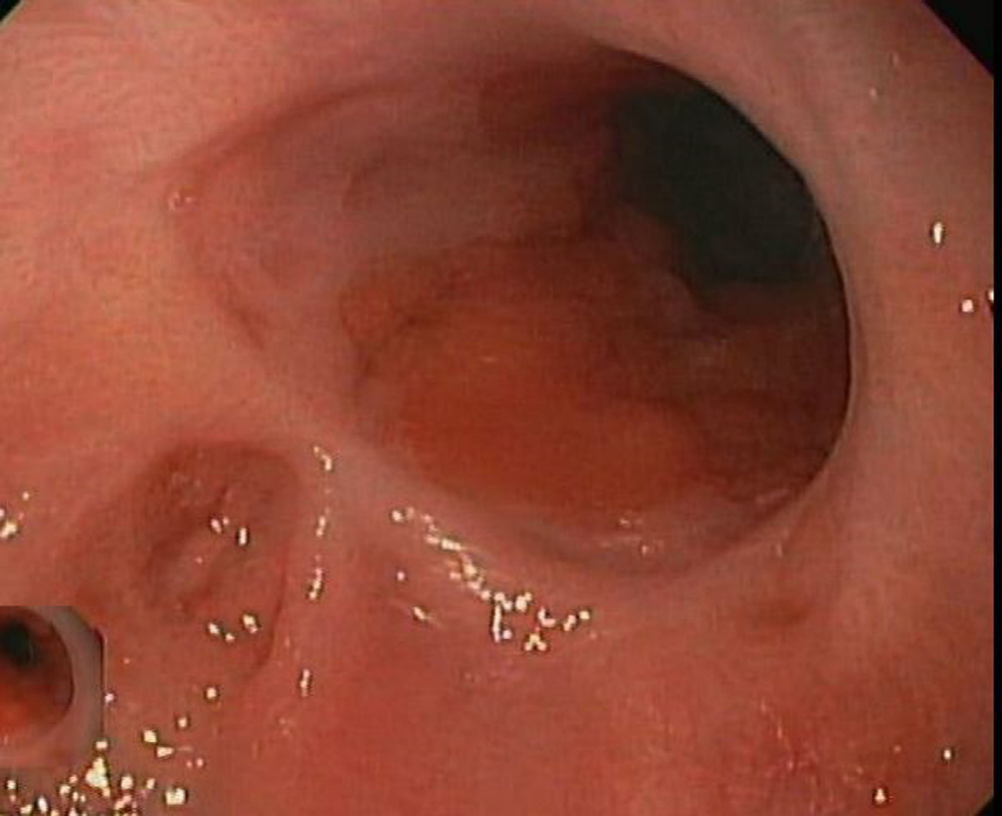
Esophageal stricture after stent removal.

A follow-up endoscopy 1 week later was performed to retrieve the FC-SEMS and showed a moderate peptic stricture that was dilated to 15 mm and subsequently 18 mm on repeat endoscopy (Figs. 8 and 9). The patient is now without dysphagia on a regular diet and remains on indefinite proton pump inhibitor therapy.

## Conclusions

We report a novel use of EndoRotor to assist in removal of a fully embedded esophageal stent. The EndoRotor provides a new technique that is a safe and effective modality for the management of embedded esophageal stents.

## Disclosure

*The authors disclosed no financial relationships.*

## Supplementary data

Video 1 Use of endoscopic morcellator to assist in removal of stent.
